# Qualitätssicherung in der Vorsorgekoloskopie in Österreich und europaweit

**DOI:** 10.1007/s41971-022-00137-w

**Published:** 2022-10-27

**Authors:** Jasmin Zessner-Spitzenberg, Elisabeth Waldmann, Monika Ferlitsch

**Affiliations:** 1 Klinische Abteilung für Gastroenterologie und Hepatologie, Univ. Klinik für Innere Medizin III, Medizinische Universität Wien, Währinger Gürtel 18–20, 7i, 1090 Wien, Österreich; 2 Arbeitsgruppe Qualitätssicherung, Österreichische Gesellschaft für Gastroenterologie und Hepatologie, Wien, Österreich; 3 Abteilung für Innere Medizin II, Gastroenterologie und Hepatologie, Evangelisches Krankenhaus Wien, Wien, Österreich

**Keywords:** Darmkrebs, Krebsfrüherkennung, Koloskopie, Qualitätssicherung in der Gesundheitsversorgung, Diagnostische Screeningprogramme, Colorectal neoplasms, Early detection of cancer, Colonoscopy, Quality assurance, health care, Diagnostic screening programs

## Abstract

Die Vorsorgekoloskopie als effizientes Tool zur Reduktion von Kolorektalkarzinominzidenz und -mortalität ist nur dann effektiv, wenn sie unter hohen Qualitätsstandards durchgeführt wurde. Die European Society for Gastrointestinal Endoscopy gibt hierbei Key Performance Measures, wie die Adenomentdeckungsrate, die Zökumerreichsrate und die Rate an adäquater Vorbereitungsqualität, vor, auf die beim Screening geachtet werden sollten. Das „Qualitätszertifikat Darmkrebsvorsorge“, das als Qualitätssicherungsprogramm auf freiwilliger Basis von der Österreichischen Gesellschaft für Gastroenterologie und Hepatologie gemeinsam mit dem Dachverband der österreichischen Sozialversicherungsträger und der Österreichischen Krebshilfe für Endoskopiker:innen in ganz Österreich ins Leben gerufen wurde, überprüft diese Qualitätsparameter. Es wird ein Darmkrebsscreening auf höchsten Standards angestrebt, um somit die besten Outcomes für Patient:innen zu erzielen. Auch europaweit ist das Interesse an einer qualitätsgesicherten Vorsorgekoloskopie groß: Viele Länder, wie z. B. die Niederlande, Norwegen und das Vereinigte Königreich haben Programme, um die Qualität des Screenings zu überwachen und zu verbessern.

## Abstract (en)

Screening colonoscopy, an efficient tool for reducing the incidence and mortality of colorectal cancer, is only effective if it is performed under high quality standards. The European Society for Gastrointestinal Endoscopy has identified the following key performance measures for screening: adenoma detection rate, cecal intubation rate, and adequate bowel preparation rate. The Quality Certificate for Colorectal Cancer Screening—which was launched as a quality assurance program on a voluntary basis by the Austrian Society of Gastroenterology and Hepatology together with the umbrella organization of Austrian social insurance carriers and the Austrian Cancer Aid for endoscopists throughout Austria—monitors these quality parameters. The aim is to achieve the highest standards of colorectal cancer screening in order to achieve the best outcomes for patients. Interest in quality-assured screening colonoscopy is also high throughout Europe: many countries, e.g., the Netherlands, Norway, and the United Kingdom, have programs to monitor and improve the quality of screening.

## Qualität in der Vorsorgekoloskopie

Qualitätssicherung in Screeningprogrammen ist eines der vorgeschriebenen Merkmale von effektivem Screening laut WHO [1]. Tatsächlich bietet eine qualitativ hochwertige Vorsorgekoloskopie einen wesentlich effektiveren Schutz gegen das kolorektale Karzinom als eine Vorsorgekoloskopie, die unter niedrigen Standards durchgeführt wurde [2]. Das Wort *qualitativ hochwertig* ist hierbei das Schlüsselwort. Um tatsächlich als Patient:in von einer Koloskopie und dem dementsprechenden Schutz vor einem kolorektalen Karzinom zu profitieren, ist eine leitlinienkonforme Qualität der Untersuchung unumgänglich. Bereits im Jahr 2010 gab die Europäische Kommission eine Leitlinie zur Qualitätssicherung der Vorsorgekoloskopie heraus [3]. Im Jahr 2012 folgte die European Society for Gastrointestinal Endoscopy (ESGE) mit einem Positionspapier zu notwendigen Standards der Qualitätssicherung [4]. In aktueller Version (2017) sind die derzeit empfohlenen „performance measures for lower gastrointestinal endoscopy“ angeführt [5]. In der Richtlinie von Kaminski et al. werden jene Parameter festgehalten, die nachgewiesenermaßen mit einem besseren Outcome der Patient:innen verbunden sind. Besonders die Adenomentdeckungsrate („adenoma detection rate“ [ADR]) war in großen Kohortenstudien durchwegs sowohl mit einer niedrigeren Inzidenz [6] als auch einer niedrigeren Mortalität des kolorektalen Karzinoms assoziiert [7]. Im Leitlinienupdate im Jahr 2017 wurde die angestrebte ADR von 20 % auf 25 % angehoben. Der Grund für die Anhebung war, dass der Cut-off-Wert der ADR von 24,6 % mit der niedrigsten Mortalität des kolorektalen Karzinoms assoziiert war [4, 8]. Die Fähigkeit, Adenome zu entdecken, ist wesentlich von der Darstellung der gesamten Kolonmukosa abhängig. Daher ist es wichtig, neben einer hohen ADR eine adäquate Vorbereitungsqualität und eine ausreichende Zökumerreichsrate zu erzielen. Dabei soll ein validiertes Scoringsystem verwendet werden (wie etwa die Boston Bowel Preparation Scale [9] oder die Aronchick Scale [10]), wobei die Vorbereitungsqualität des beurteilten Kolons zumindest mittelmäßig sein soll [6, 11]. Ebenfalls sollten Endoskopiker:innen nach einer hohen Rate an Koloskopien streben, bei der bis in das Zökum vorgespiegelt wird, um mit hoher Konfidenz das gesamte Kolon inklusive eventueller Polypen beurteilen zu können. Eine niedrige Zökumerreichsrate ist mit einer höheren Inzidenz an kolorektalen Karzinomen assoziiert, sowohl distal als auch proximal [12]. Derzeit wird ein Mindeststandard der Zökumerreichsrate von ≥ 90 % angegeben, wobei eine Rate von ≥ 95 % optimal ist [13]. Um zu gewährleisten, dass Polypen komplett abgetragen werden, gibt die ESGE eine leitlinienkonforme Abtragungsrate von mindestens 80 % vor. Das bedeutet, dass Polypen < 10 mm mit der kalten Schlinge und ≥ 10 mm mit der heißen Schlinge abgetragen werden sollen [14]. Eine inadäquate Abtragungstechnik von Polypen ist für bis zu 25 % der kolorektalen Karzinome nach Screeningkoloskopie (= „post-colonoscopy colorectal cancer“ [PCCRC]) verantwortlich [15].

Obwohl die Koloskopie sehr sicher ist, können Komplikationen auftreten. Studien geben beispielweise eine Komplikationsrate von etwa 0,3–0,5 pro 1000 Untersuchungen an [16, 17]. Die ESGE gibt vor, Komplikationen, die während der Untersuchung auftreten, sowie Events, die nach einer Koloskopie auftreten, wie etwa Spitalsaufenthalte oder die 30-Tage-Mortalität, zu dokumentieren [13].

Um eine hohe Patient:innencompliance zu erzielen, ist es notwendig, Schmerzen und Unannehmlichkeiten während einer Koloskopie zu minimieren. Auch wenn es derzeit keinen Standard zur Patient:innenzufriedenheit gibt, können effiziente Sedierungspraktiken bei der Koloskopie helfen, das Empfinden der Koloskopie zu verbessern [18].

Zu guter Letzt ist nach der Vorsorgekoloskopie das korrekte Management mit etwaigen Follow-up-Koloskopien essenziell. Nur bei Einhalten der adäquaten Nachsorge nach Polypektomie können eventuell neu gewachsene oder inkomplett resezierte Polypen entfernt werden. Laut ESGE sollten Patient:innen, die Hochrisikopolypen haben, eine erneute Koloskopie in 3 Jahren bekommen, eine weitere Koloskopie in 10 Jahren ist für Patient:innen ohne Hochrisikopolypen sinnvoll. Die Definition der Hochrisikopolypen wurde im Jahr 2020 aktualisiert [19]. Somit soll eine Nachsorge nur bei Patient:innen stattfinden die

- mindestens ein Adenom mit hochgradiger Dysplasie oder
- mindestens ein Adenom ≥ 10 mm oder
- ≥ 5 Adenome oder
- mindestens einen serratierten Polypen mit Dysplasie oder
- mindestens einen serratierten Polypen ≥ 10 mm

aufweisen.

## Das Qualitätszertifikat Darmkrebsvorsorge

Das Qualitätszertifikat Darmkrebsvorsorge ist ein von der Österreichischen Gesellschaft für Gastroenterologie und Hepatologie gemeinsam mit dem Dachverband der Österreichischen Sozialversicherungsträgern und der Österreichischen Krebshilfe (Leitung: a.o. Univ. Prof. Dr. Monika Ferlitsch) ins Leben gerufenes freiwilliges Qualitätssicherungsprogramm für österreichische Ärzt:innen, die Vorsorgekoloskopien durchführen. Seit der Gründung im Jahr 2007 ist das Projekt dabei stetig gewachsen. Während in den Jahren 2007–2009 und 2010–2011 217 endoskopierende Stellen verzeichnet waren, waren in der Periode 2012–2013 223, in der Periode 2014–2015 218, in der Periode 2016–2017 211 und in der Periode 2018–2019 226 Stellen zertifiziert. Mit Erhebung 12/2021 verzeichnet das Programm 239 teilnehmende endoskopierende Stellen und repräsentiert damit ca. 45 % aller Endoskopiestellen Österreichs. Seit Projektstart im Jahr 2007 wurden über 447.857 Befunde von den zertifizierten Ärzt:innen übermittelt, im Jahre 2021 waren es 39.432, ein trotz SARS-CoV-2-Pandemie wieder stabiler Trend. Eine Studie, die die durchgeführten Koloskopien pro Kalenderwoche 2019 zu 2020 verglich, konnte zeigen, dass kurz nach Verlautbarung der ersten COVID-19-Restriktionen die Anzahl der übermittelten Befunde in derselben Kalenderwoche im Jahr um bis zu 92 % geringer war [20].

**Figure 1:**
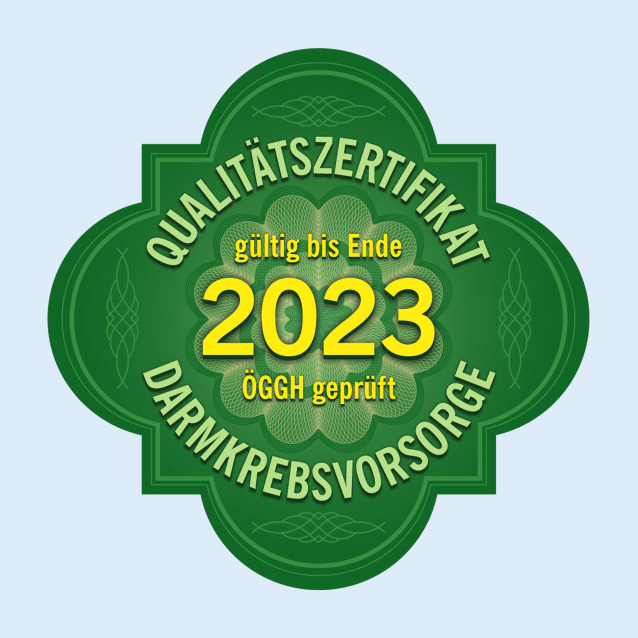

Ziel des Programms ist es, endoskopierende Ärzt:innen, die sich an internationale Qualitätsstandards halten, mit dem offiziellen „Qualitätszertifikat Darmkrebsvorsorge“ auszuzeichnen (Abb. 1). Zu den Anforderungen des Programms zählen die Bereitschaft, regelmäßig die Befunde aller durchgeführten Vorsorgekoloskopiebefunde zu übermitteln, Hygienestandards einzuhalten und sich einer regelmäßigen Stichprobenkontrolle der Befunddatenqualität zu unterziehen. Eine Teilnahme am Qualitätszertifikat Darmkrebsvorsorge hat für Teilnehmer:innen viele Vorteile: So erfolgt bei Zertifizierung eine Nennung auf der offiziellen Website des Qualitätszertifikats und Teilnehmer:innen werden in das Verzeichnis der empfohlenen Ärzt:innen bzw. Institutionen der Österreichischen Krebshilfe aufgenommen. Ordinationen profitieren zusätzlich von einer Rückerstattung der jährlich durchgeführten Hygieneüberprüfung.

Die Qualitätssicherung des Zertifikats lebt von einer regelmäßigen Prüfung ihrer Standards. Teilnehmer:innen werden demnach auch auf Performanceparameter, angelehnt an die Leitlinie des ESGE, geprüft. So zählen eine ausreichend hohe Adenomentdeckungsrate von 25 %, eine adäquate Vorbereitungsqualität in ≥ 90 % (Ziel 95 %) der Untersuchungen, eine Zökumerreichsrate von ≥ 90 % (Ziel 95 %) und eine korrekte Polypenabtragungstechnik in ≥ 80 % zu den vom Projekt erstrebten Standards.

**Figure 2:**
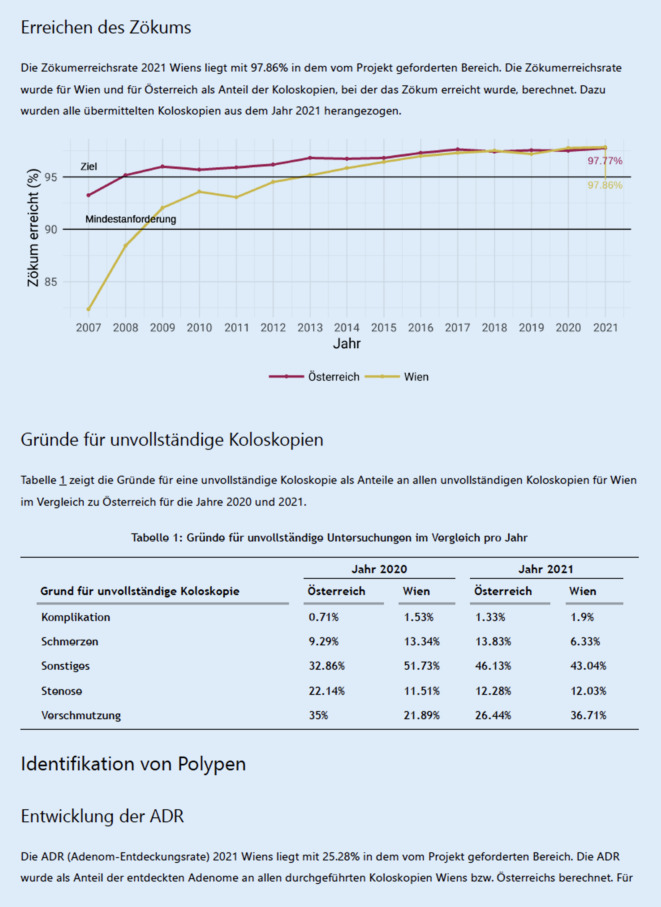

Zu einer effektiven Qualitätssicherung gehört nicht nur ein regelmäßiges Audit der Qualitätsparameter, sondern auch ein Feedback an die teilnehmenden Endoskopiker:innen. Im Rahmen eines 2‑mal jährlich ausgesendeten Benchmarkingberichts bekommen Teilnehmer:innen eine Rückmeldung über die Qualitätsparameter der von ihnen durchgeführten Koloskopien und wie ihre Performance im Vergleich zu allen Teilnehmer:innen in Österreich ist. Ein Auszug aus einem Benchmarkingbericht zeigen Abb. 2.

**Figure 3:**
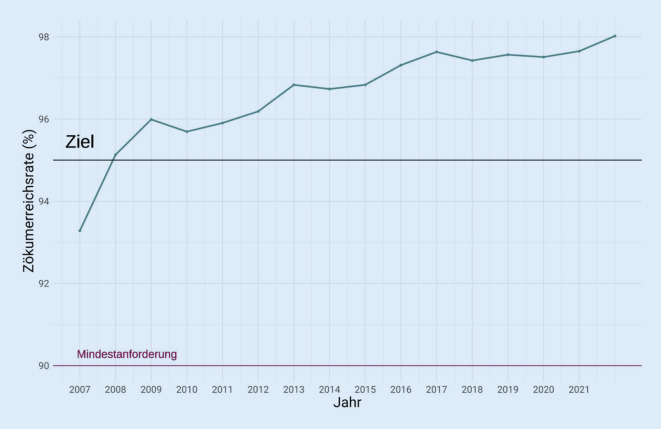

**Figure 4:**
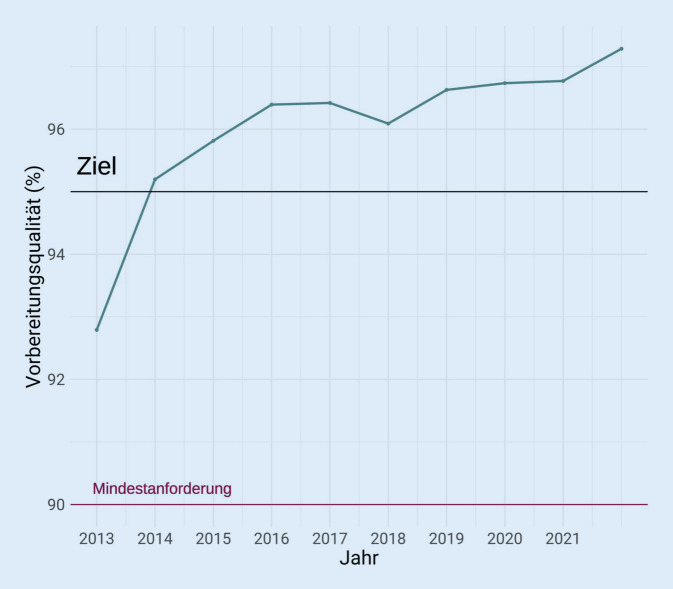

Die Qualität der Vorsorgekoloskopien, die im Rahmen des Qualitätszertifikats durchgeführt werden, zeigt insgesamt eine stetige Verbesserung. Während die durchschnittliche Zökumerreichsrate zu Beginn der Datenaufzeichnung 2007 noch unter 95 % lag, ist sie mittlerweile auf 98 % im Jahr 2021 angestiegen (Abb. 3). Ein ähnlicher Trend lässt sich bei der Rate an adäquater Vorbereitungsqualität feststellen. Mehr als 97 % der durchgeführten Vorsorgekoloskopien werden bei einer ausgezeichneten, guten oder mittelmäßigen Vorbereitungsqualität durchgeführt (Abb. 4).

**Figure 5:**
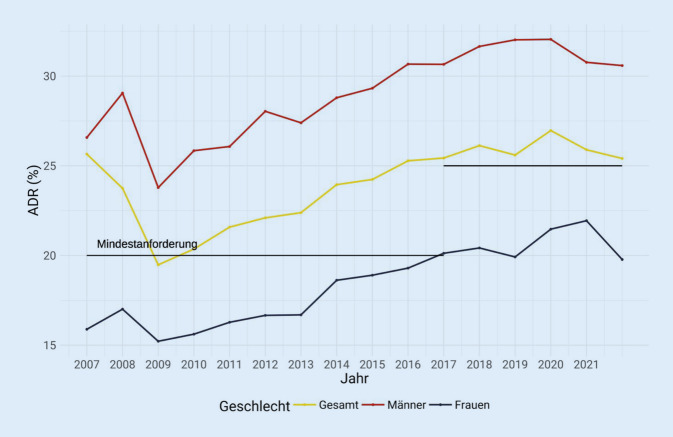

Bei der Adenomentdeckungsrate ist seit Start des Projekts ein positiver Trend zu beobachten (Abb. 5).

In Österreich wird derzeit ein opportunistisches Darmkrebsscreening angeboten. Im Gegensatz zu einem populationsbezogenen Screening, im Rahmen dessen die Zielbevölkerung eine Einladung (idealerweise bereits mit Termin), am Screening teilzunehmen, erhält, erfolgt die Teilnahme in einem opportunistischen Screeningprogramm auf Eigeninitiative bzw. durch Zuweisung von einer/m Allgemeinmediziner:in oder Fachärzt:in. Es ist somit wesentlich schwieriger, die Teilnahmeraten sowie die Qualität der durchgeführten Untersuchungen zu erheben.

Obwohl sich die Teilnehmer:innen am Qualitätszertifikat Darmkrebsvorsorge dazu verpflichten, Befunde aller von ihnen durchgeführten Koloskopien zu übermitteln, vorausgesetzt es wird davor die Einverständnis der Patient:innen eingeholt, ist es unmöglich, die Vollständigkeit der Befundübermittlung zu überprüfen. Somit ist ein gewisser Reporting Bias nicht mit letzter Sicherheit auszuschließen.

Um optimale Patient:innenversorgung sowie wirtschaftliche Effektivität zu ermöglichen, wäre die Implementierung eines verpflichtenden Qualitätssicherungsprogramms idealerweise im Rahmen eines populationsbezogenen Screeningprogramms erstrebenswert.

### Koloskopiequalitätsforschung in Österreich

Die Initiative des Qualitätszertifikats Darmkrebsvorsorge dient nicht nur der Erfassung, Beurteilung und Sicherung anerkannter Qualitätsstandards; die Datenerfassung ist auch Grundlage der Qualitätsforschung. Dank der hochqualitativen Datenlage konnte gezeigt werden, dass pro Prozentpunkt höherer ADR ein 3 % niedrigeres Risiko bestand, ein kolorektales Karzinom nach einer Vorsorgekoloskopie zu entwickeln [21]. Zudem wurde gezeigt, dass eine niedrigere ADR ein genauso großer Risikofaktor für das Auftreten eines kolorektalen Karzinoms nach einer Vorsorgekoloskopie ist wie das Vorhandensein von Hochrisikopolypen (≥ 3 Adenome, (tubulo)villöse Adenome, Polypen ≥ 10 mm, Polypen mit hochgradiger Dysplasie; [21]). Was die Mortalität am kolorektalen Karzinom nach einer Screeningkoloskopie betrifft, zeigt sich ein ähnlicher Trend. Das Risiko, an einem kolorektalen Karzinom zu versterben, war bei Hochrisikopatient:innen fast doppelt so hoch, wenn der/die jeweilige Endoskopiker:in eine ADR < 25 % bei der Untersuchung hatte, im Vergleich zu Hochrisikopatient:innen, die von einem/einer Endoskopiker:in mit einer ADR ≥ 25 % untersucht wurden [22].

## Europaweite Qualitätssicherung

Obwohl die Qualitätssicherung essenziell für die Effektivität eines Screeningprogramms ist, ist das Qualitätsmonitoring in Europa sehr heterogen. Eine europaweite Studie der European Colonoscopy Quality Investigation Group an 52 Institutionen und 92 Endoskopiker:innen ergab, dass lediglich 29 % der Institutionen die ADR aufzeichnen, die Zökumerreichsrate 62 % und die Rate der adäquaten Vorbereitungsqualität nur 56 % [23]. Um die Qualität der Koloskopie zu sichern, haben sich in den verschiedenen Ländern unterschiedliche Ansätze entwickelt. Norwegen, eine der vormals führenden Nationen in der Inzidenz des kolorektalen Karzinoms, hat ein nationales Qualitätssicherungsprogramm eingeführt. Dieses umfasst Krankenhäuser als teilnehmende Institutionen und wurde 2003 auf dem Konzept der Norwegian-Colorectal-Cancer-Prevention(NORCCAP)-Studie entwickelt. In Ländern wie den Niederlanden oder dem Vereinigten Königreich ist das Qualitätsmonitoring eng an das organisierte Screeningprogramm geknüpft.

## Screeningprogramme in Europa

Die sinkende Inzidenz und Mortalität des kolorektalen Karzinoms in Europa lässt sich zu einem großen Teil auf die Einführung von Screeningprogrammen zurückführen [24]. Deutschland, die Tschechische Republik und Polen haben seit Anfang der 2000er-Jahre auf ein primäres Koloskopiescreening gesetzt. Das bedeutet, dass die Zielpopulation ein Screening mittels einer Koloskopie erhält, ohne dass ein Test auf Blut im Stuhl vorgeschaltet wird. In Österreich wird sowohl ein Stuhltest auf okkultes Blut als auch ein primäres Koloskopiescreening angeboten. In den übrigen europäischen Nationen wird weitgehend ein Screening mittels Stuhltest durchgeführt. Hierbei hat sich in den letzten Jahren großteils der Fecal Immunochemical Test (FIT) gegenüber dem Guiac Fecal Occult Blood Test (gFOBT) durchgesetzt [25]. Welche die effektivste Methode des Screenings ist, ist umstritten. Im Rahmen von rezenten randomisierten populationsbasierten Studien konnten höhere Teilnahmeraten bei stuhltestbasierten Screeningmethoden gezeigt werden [26, 27]. Die Vorsorgekoloskopie bietet hingegen den Vorteil, dass potenziell vorhandene Läsionen bereits in einem früheren Stadium entdeckt und im selben Untersuchungsgang auch abgetragen werden können [28, 29].

Die größte Reduktion an Kolorektalkarzinominzidenz und -mortalität konnte in Ländern erreicht werden, die früh (bereits vor 2000) Screeningprogramme eingeführt haben: Eine große Studie, die in *Lancet Oncology* publiziert wurde, zeigte hierbei, dass vor allem Deutschland, die Tschechische Republik und Österreich starke Rückgänge dank ihrer Screeningprogramme verzeichnen konnten [30]. Zeitgleich sind diese Länder auch jene, die ein primäres Koloskopiescreening anbieten. Welche Methode (FIT-Screening vs. primäres Koloskopiescreening) tatsächlich die effektivere hinsichtlich Mortalitätsreduktion ist, ist Gegenstand aktueller randomisierter Studien [26].

So heterogen wie die implementierten Screeningprogramme in Europa sind auch die jeweiligen berichteten Qualitätsparameter. Die größten Unterschiede sind naturgemäß in der ADR zu finden: Aufgrund der höheren Vortestwahrscheinlichkeit, Adenome bei Patient:innen, die einen FIT-Test hatten, zu entdecken, ist die ADR in FIT-Screening-Ländern im Durchschnitt um 15 % höher als in jenen Ländern, wo die Koloskopie als primäre Screeningmethode angeboten wird [31]. Ein ähnlicher Trend verzeichnet sich bei den Raten der entdecken fortgeschrittenen Adenome („advanced adenoma dection rate“ [AADR]), wobei diese Differenz kleiner ist. Tab. 1 zeigt eine Übersicht der einzelnen berichteten Qualitätsparameter in verschiedenen europäischen Ländern. Die Zökumerreichsrate liegt europaweit, bis auf einzelne Ausnahmen, weitgehend über den von der ESGE geforderten 90 %. Ähnlich hoch sind die Raten an Koloskopien, bei denen Patient:innen eine ausreichende Vorbereitungsqualität vorweisen. In den meisten europäischen Ländern ist zudem erfreulich, dass die Koloskopie sicher ist. Die Rate an Komplikationen (Blutungen, Perforationen, kardiovaskuläre Events) schwankt zwar zwischen den Nationen, erreicht jedoch niemals > 1 %.

**Table 1:** 

Land	ADR (%)	AADR (%)	SR (%)	CIR (%)	CR (%)	ABPR (%)	REF
Deutschland	31,3(m), 20,1 (f)	9,0(m), 5,2(f)	92,85	98	0,31^b^	98,6^c^	[17, 32]
UK	52,9(m) 36,5(f)	NA	94,6	92,5	0,10^b^	97^c^	[35–37]
Italien^a^	15,9(m), 8,4(f)	6,4(m), 3,0(f)	55,1	80,7	0,24^b^	93,7^c^	[32, 38–40]
Polen	35,6(m), 21,0(f)	7,7(m), 3,8(f)	45,9	97,8	0,12^b^	94,6^c^	[32, 41]
Norwegen	34,3(m), 20,9(f)	10,5(m) 5,6(f)	10,8	96,3	0,15^b^	93,7^c^	[41, 42]
Österreich	33,76(m), 23,02 (f)	7,6(m), 4,0(f)	87,6	98,7	0,2	92,4^c^	[43–45]
Niederlande	31,8	11,5^d^	90,0	98,5	0,41^b^	93,7^c^	[41, 46, 47]
Frankreich	38,8(m) 22,1(f)	16,2	95	89,7	0,35	93,1	[48–50]
Portugal	44,4	4,4	25	92	0,12	95,1	[51, 52]

*ADR* „adenoma detection rate“, *AADR* „advanced adenoma detection rate“, *CIR* „cecal intubation rate“, *CR* „complication rate“, *ABPR* „adequate bowel preparation rate“, *SR* Sedierungsrate, *ZIR* Zökumerreichsrate, *KR* Komplikationsrate, *VQ* Vorbereitungsqualität, *REF* Referenzen, *m* Männlich, *f* Weiblich

^a^ADR für Sigmoidoskopie, Zökumerreichsrate von Beobachtungsstudien

^b^Komplikationsrate von Blutungen

^c^Ausgezeichnete/gute/mittelmäßige Vorbereitungsqualität

^d^Pro 1000 gescreente Personen

## Ausblick

Im Jahr 2020 jährte sich die Deklaration des europäischen Parlaments zur Bekämpfung des kolorektalen Karzinoms zum 10. Mal [32]. Seit die Deklaration in vielen europäischen Ländern implementiert wurde, rückt die Sicherung der Qualitätsstandards der Vorsorgekoloskopie zunehmend in den Fokus. Nicht nur das regelmäßige Monitoring, sondern auch die regelmäßige Anpassung der Qualitätsparameter auf Basis der wissenschaftlichen Erkenntnisse ist ein laufender Prozess. Die kommenden Monate und Jahre werden voraussichtlich wesentliche Neuerungen hinsichtlich jener Qualitätsparameter bringen, die aktuell noch keinen Qualitätsstandard festgelegt haben (z. B. Patient:innenzufriedenheit). Zudem wird die Empfehlung hinsichtlich der Qualitätsparameter in Zukunft weiter angepasst werden. Die Adenomentdeckungsrate wird vermutlich einen eigenen Standard für Populationen mit FIT/gFOBT erhalten.

Eine weitere wünschenswerte Neuerung betrifft individualisierte Vorsorgeempfehlungen. Die ESGE empfiehlt derzeit eine ADR ≥ 25 % für alle Personen, die sich einer Vorsorgekoloskopie unterziehen, obwohl bekannt ist, dass Männer doppelt so häufig und in einem 10 Jahre jüngeren Alter als Frauen Adenome entwichen [33]. Auch wird sich zeigen, ob die Charakteristika der untersuchten Population (hohe Prävalenz von Risikofaktoren für Polypen wie hoher BMI oder Diabetes mellitus) zu einem personalisierten ADR-Ziel führen werden.

Die ESGE gibt derzeit noch keinen Standard für die Messung der Patient:innenzufriedenheit vor. Validierte Fragebögen zur Erfassung der Zufriedenheit existieren bereits. Studien, die beleuchten, ob die Messung der Zufriedenheit auch in eine höhere Compliance übertragbar ist, sind derzeit ausständig [13, 34].

Zudem werden Ergebnisse großer randomisierter Head-to-head-Studien zur (Kosten‑)Effektivität verschiedener Screeningmethoden (primäres Koloskopiescreening, FIT-Screening) erwartet.

## Fazit für die Praxis

„What gets measured gets managed“ – so der österreichstämmige US-Ökonom Peter Drucker. Tatsächlich können wir uns nur verbessern, wenn wir den Ist-Zustand festhalten und beurteilen. Die Implementierung eines landesweiten populationsbezogenen Screeningprogramms mit Einladung ist eine Voraussetzung, um dieses Ziel zu erreichen. Die Qualitätssicherung in der Vorsorgekoloskopie hat Zukunft – in Österreich und europaweit.
